# Supplementary material for: Semantic micro-contributions with decentralized nanopublication services
Source: PeerJ Comput Sci. 2021 Mar 8;7:e387. doi: 10.7717/peerj-cs.387 (PMC7959648; doi:10.7717/peerj-cs.387)
Supplement: Supplemental Information 1 — Contains the code and data that was used and generated for the performance evaluation and the usability study. [file peerj-cs-07-387-s001.zip › nanobench/src/main/java/org/petapico/nanobench/NanopubItem.html]

—
 —
 —
