# Supplementary material for: Semantic micro-contributions with decentralized nanopublication services
Source: PeerJ Comput Sci. 2021 Mar 8;7:e387. doi: 10.7717/peerj-cs.387 (PMC7959648; doi:10.7717/peerj-cs.387)
Supplement: Supplemental Information 1 — Contains the code and data that was used and generated for the performance evaluation and the usability study. [file peerj-cs-07-387-s001.zip › nanobench/src/main/java/org/petapico/nanobench/OrcidLinkingPage.html]

nanobench


## Linking your ORCID Account

To prove that you are the one who has control of the given ORCID account, you can link your introduction nanopublication from your public ORCID profile.

To do this, login to ORCID and go to your ORCID record.
There, click on the small pencil icon next to "Websites" on the left side bar:

Click then on the plus icon at the bottom of the internal window that comes up:

Two new text fields show up. In the first one, labeled "Description", put `intro-nanopub`.
In the second one, labeled "URL", paste the URL of your introduction nanopublication, which is
.

Then press "Save changes".
As a last check, you only need to ensure that the visibility of your profile is set to "Everyone" in
your ORCID account settings:

Finally, you can go back to your profile and press 'retry' to check your ORCID linking again.
