# Supplementary material for: Semantic micro-contributions with decentralized nanopublication services
Source: PeerJ Comput Sci. 2021 Mar 8;7:e387. doi: 10.7717/peerj-cs.387 (PMC7959648; doi:10.7717/peerj-cs.387)
Supplement: Supplemental Information 1 — Contains the code and data that was used and generated for the performance evaluation and the usability study. [file peerj-cs-07-387-s001.zip › nanobench-usability-eval/form.html]

Nanobench Usability Feedback   

Nanobench Usability Feedback

Thank you for having tried out Nanobench. Help us assess the tool's usability by answering the questions below. If you need to refresh your memory, see the screenshot below (and more here: https://github.com/peta-pico/nanobench) or run the tool once more.  
  
The data of this questionnaire are collected in a fully anonymous way.

\* Required

This questionnaire is about the Nanobench tool that you previously used:

General Questions

Have you published RDF / Linked Data before? \*

Yes

No

If yes, was it easier or harder to publish RDF / Linked Data with Nanobench, as compared to how you previously did it?

nanobench was harder

1

2

3

4

5

nanobench was easier

Have you digitally signed RDF / Linked Data before? \*

Yes

No

If yes, was it easier or harder to digitally sign RDF / Linked Data with Nanobench (which happens each time you publish a nanopublication), as compared to how you previously did it?

nanobench was harder

1

2

3

4

5

nanobench was easier

Tell us whether you agree with the statements below ("the system" = nanobench)

I think that I would like to use this system frequently. \*

strongly disagree

1

2

3

4

5

strongly agree

I found the system unnecessarily complex. \*

strongly disagree

1

2

3

4

5

strongly agree

I thought the system was easy to use. \*

strongly disagree

1

2

3

4

5

strongly agree

I think that I would need the support of a technical person to be able to use this system. \*

strongly disagree

1

2

3

4

5

strongly agree

I found the various functions in this system were well integrated. \*

strongly disagree

1

2

3

4

5

strongly agree

I thought there was too much inconsistency in this system. \*

strongly disagree

1

2

3

4

5

strongly agree

I would imagine that most people would learn to use this system very quickly. \*

strongly disagree

1

2

3

4

5

strongly agree

I found the system very cumbersome to use. \*

strongly disagree

1

2

3

4

5

strongly agree

I felt very confident using the system. \*

strongly disagree

1

2

3

4

5

strongly agree

I needed to learn a lot of things before I could get going with this system. \*

strongly disagree

1

2

3

4

5

strongly agree

Further Feedback (optional)

You can write any further feedback here:

Your answer

That's all. Thank you for your help!

And feel free to keep using nanobench.

Submit

Never submit passwords through Google Forms.

This content is neither created nor endorsed by Google. Report Abuse - Terms of Service - Privacy Policy

Forms
