# Supplementary material for: Semantic micro-contributions with decentralized nanopublication services
Source: PeerJ Comput Sci. 2021 Mar 8;7:e387. doi: 10.7717/peerj-cs.387 (PMC7959648; doi:10.7717/peerj-cs.387)
Supplement: Supplemental Information 1 — Contains the code and data that was used and generated for the performance evaluation and the usability study. [file peerj-cs-07-387-s001.zip › nanopub-services-eval/eval/query-chart.pdf]

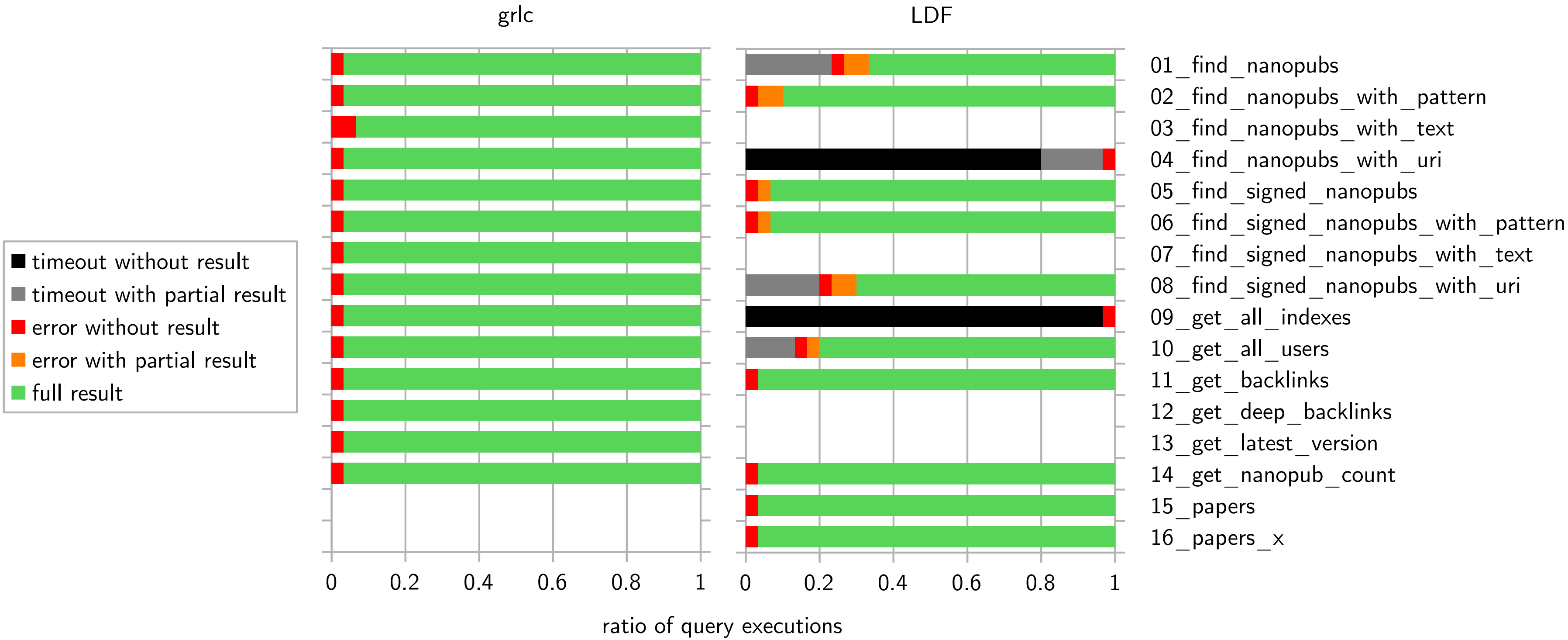
grlc
LDF
timeout without result
timeout with partial result
error without result
error with partial result
full result
01_find_nanopubs
02_find_nanopubs_with_pattern
03_find_nanopubs_with_text
04_find_nanopubs_with_uri
05_find_signed_nanopubs
06_find_signed_nanopubs_with_pattern
07_find_signed_nanopubs_with_text
08_find_signed_nanopubs_with_uri
09_get_all_indexes
10_get_all_users
11_get_backlinks
12_get_deep_backlinks
13_get_latest_version
14_get_nanopub_count
15_papers
16_papers_x
0
0.2
0.4
0.6
0.8
1
ratio of query executions
